# Impact of Hypertension and Physical Fitness on SARS-COV-2 and Related Consequences. (Possible Mechanisms with Focusing on ACE2)

**DOI:** 10.22088/cjim.13.0.148

**Published:** 2022

**Authors:** Mehdi Kushkestani, Mohsen Parvani, Mahsa Moghadassi, Yaser Kazemzadeh, Kiandokht Moradi

**Affiliations:** 1 Faculty of Physical Education and Sport Sciences, Allameh Tabataba'i University, Tehran, Iran; 2 Department of Exercise Physiology, Islamic Azad University, Tehran North Branch, Tehran, Iran; 3 Department of Exercise Physiology, Islamshahr branch, Islamic Azad University, Islamshahr, Iran

**Keywords:** SARS-CoV-2, Hypertension, Exercise, Angiotensin-converting enzyme 2, Physical Fitness, Renin-Angiotensin System

## Abstract

Hypertension disease as an absolute risk factor of Covid-19 disease has been well-proven in recent evidence. The factors such as the use of antihypertensive drugs, protein expression, and compensatory axes resulted in hypertension disease playing very important roles in the occurrence of this problem. In this review study, we first attempted to investigate the higher chance reason for Covid-19 disease in people with high blood pressure; then we examined the related mechanisms, and finally, we reported the differences and similarities between people with high blood pressure and athletes. All in all, we concluded that people who exercise regularly, the same as hypertensive patients (Compensatory mechanism) are more susceptible to COVID-19 infection due to the high concentration of ACE2 (Physiological mechanism) caused by exercise adaptation, but for the low level of ANG2 (Systematic and gene expression) these individuals (Active subjects) indicate fewer complications and severity symptoms of COVID-19 such as dyspnea, hospitalization and, heart disease compared with hypertensive patients.

Coronavirus 2019 is an infected illness that was first identified in China in December 2019 and included a very high prevalence rate all around the world and has become a pandemic phenomenon (1–3). This virus is induced by severe acute respiratory syndrome coronavirus 2 (SARS-CoV-2), which is associated with mild to moderate respiratory distress and generally improves without the need for specific intervention. Older adults with comorbidity, such as cardiac disorder, type 2 diabetes, and chronic respiratory disease, experience different conditions if they infected to them (4,5). Clinical and epidemiological symptoms include acute onset of pyrexia and cough or acute onset (3 or more symptoms) of pyrexia, cough, fatigue, headache, myalgia, throat ache, coryza, anorexia / qualm / vomiting, flux, and moral state change. Generally, people have mild symptoms, but some of the cases are involved in acute respiratory distress syndrome (ARDS), which may be caused by septic shock, cytokine storm, and multi-organ failure (6–8). The COVID-19 virus is produced during coughing, sneezing, and small aerosol particles and is transferred by breathing, talking to an infected person, and possibly through infected surfaces (This case has not yet been proven) (1). In moderate and mild cases, the infected person stays infected for 7-12 days and in more severe cases for up to two weeks (4). Based on the report of the World Health Organization (WHO), as of October 9, 2020 there have been 36,616,555 reported cases of COVID-19 worldwide, including 1,063,429 deaths (9).

**Covid-19 Structure and Connection to the Host Cells: **Applying computer designing, researchers have observed that SARS-CoV-2 and SARS-CoV spike protein (SP) have similar 3-D anatomy in the receptor-binding domain which keeps van der Waals forces (10,11). SARS-CoV SP has a powerful binding affinity to humans (Angiotensin-Converting Enzyme 2), based on biochemical and crystal structure lysis studies (12). Contrary to SARS-CoV, SARS-CoV-2 had an enormously bigger universal outbreak and has influenced higher subjects (13). SARS-CoV-2 infection depends on transmembrane serine protease (TMPRSS2) of cell for SARS-CoV-2 SP and the host cell ACE2 for entrance to body.

(11). Additional examination also recommended that SARS-CoV-2 distinguishes ACE2 at the cell surface more effectively than SARS-CoV, strengthening the SARS-CoV-2 capability to transfer from an individual to others (14). Wrapp et al. newly presented the Cryo-EM anatomy of the virus SP, the identified ligand of ACE2, and reported stronger connection of ACE2 for SARS-CoV-2 than other SARS-CoVs (15). Also, the SARS-CoV-2 SP was predicted to have a potent attachment to ACE2 in humans. Particularly, from the point of view, that enhanced expression of the ACE2 receptor, may make SAR-CoV2 more infective via rising viral load, morbidity, and mortality (16). But, interestingly Endocytosis of the SARS-CoV-2–ACE2 complex, as well as viral-induced ACE2 cell surface shedding and ACE2 downregulation, all may lead to diminished ACE2 expression and activity in infected cells (11,17). 

**ACE2/ACE Ratio: **Contrary to ACE, which is largely expressed in various organs, expression of ACE2 is limited to the kidney, endothelial cells of respiratory tissues, and cardiac venues. It’s well established that ACE2 has been a potential therapeutic goal in curing blood pressure and cardiovascular diseases (18–20). Recent investigations have also confirmed the influential role of ACE2 in balancing of the RAS system. Interestingly, in cardiac cells, ACE2 can be greater than ACE in organizing regional expression of Ang 1–7 and Ang II, and finally equivalence of RAS system. For instance, it has been determined that deficit in ACE2 levels leads to enhanced Ang II expression and circulation and thus lowered Ang 1–7 levels (21,22). The high catalytic efficiency of ACE2 for the formation of (ANG)-1-7 from ANG II recommends an important function of ACE2 in limiting ANG II accumulation, while at the same time heightening ANG-1-7 formation (23). 

**Susceptibility to** **SARS-CoV-2** **in Hypertensive Patients: **COVID-19 related comorbidity and fatality expand extremely with age and co-existing peace situations, such as heart diseases and cancer, and while most infected subjects meliorate, even healthy and young subjects may suddenly surrender to COVID-19 (24). Furthermore, some researchers discovered that hypertension has a hazard ratio of 1.70 for mortality and about 1.80 for acute respiratory distress syndrome (ARDS) in SARS-COV2 infected cases (25). Besides, Zhou and colleagues reported hypertension to have a hazard ratio of 3.05 for in-hospital death in 191 SARS-COV2 infected cases (26). Moreover, in all of these studies, subjects with severe COVID-19 were older—age 65 years and higher—making it challenging to separate the impression of age from comorbid conditions, such as hypertension. As long as the finding of more sophisticated analyses that account for age, comorbid conditions, baseline medication use, and other possible confusing factors become available, one cannot deduce that hypertension is an autonomous risk factor for SARS-CoV-2 penetration or more-severe COVID-19 (27). One of the frequently cited articles about COVID-19 proposes that people with CVD, type 2 diabetes mellitus, hypertension, or, those treated with ACE2 enhancing medications, are at larger hazard for intensive SARS-COV2 putridity and thus must be managed for ACE2-modulating drugs, including ACE inhibitors and angiotensin II type-I receptor blockers (ARBs) (28). 

On the other hand, in one cross-sectional study of children who were hypertensive, both primary hypertension and renovascular hypertension were linked with higher level of Ang-(1–7) concentrations compared with normotensive subjects. Therefore, the bulk of clinical and bestial evidences support the notion of this compensatory boost in ACE2/Ang-(1–7) expression with age raising and in the presence of variant diseases such as CVD, type 2 diabetes mellitus and hypertension (29,30). 

**The Reduction of ACE2 Expression in Infected Cells and Consequences: **It has reported that the attachment of SARS-COV2 to the binding site of ACE2 decreases ACE2 gene expression as well as, the ACE2-Ang1-7-Mas receptor axis, approximately over-activating the ACE-Ang II-AT1 (31) receptor pathway; so we can hypothesize that:

1. As with SARS-CoV, absence of ACE2 expression and activity could enhance native Ang II levels in the lungs and bring about COVID-19 acute lung injury. Reduction of ACE2 expression could raise local Ang II concentration in the lungs and cause COVID-19 acute lung inflammation, injury, and fibrosis (32,33). 

2. Furthermore, Data from the original SARS-CoV epidemic study reported that COVID-19 can lead to ACE2-dependent infection of the myocardium, inducing diminished cardiac ACE2 expression, promoting acute heart damage (34).

Eventually, regarding the recent reports about SARS-CoV-2 in hypertensive subject, the crucial roles of the RAS system especially ACE2 in SARS-CoV-2 related outcomes and mortality, as well as some similarities in athletes and hypertension subjects concerning gene expression of ACE2, the purpose of this study was to investigate of some common and adverse mechanisms between people who perform exercise and do physical activity regularly and hypertensive patients about susceptibility to SARS-CoV-2 as well as its complications.

**Data Source and Search: **We administered a vast probe of three databases, Google Scholar, EMBASE, PubMed/MEDLINE (Before, October 10. 2020). Search Keywords included: “SARS-COVID-2”, “COVID19 death”, “Exercise and SARS-COVID-2”, and “RAS System and Exercise” [MESH]. We also searched for any papers that were published in English. Besides, we manually researched references of the selected papers for additional related papers. 

**Inclusion and Exclusion Criteria: **In this narrative review, we included articles that met the following inclusion criteria: epidemiological reports concerning COVID-19 in hypertensive subjects (2) the mechanism of COVID-19 in hypertensive patients, (3) effects of exercise on the RAS system in COVID-19 article. Our exclusion criteria were: (1) case research, (2) complementary articles that are published in English, (3) correspondence pieces, (4) area level studies, and (5) retracted papers. Furthermore, we proposed to minimize the probability of including subjects from similar people twice when exploring one result.

**Data Screening and Extraction: **Two researchers separately riddled captions, abstracts and, full-text papers reporting potentially qualified researches. Datum extraction was duplicated for whole articles by a well-established scholar. 97 articles (original and review) were deemed eligible concerning our review criteria. We divided all eligible researches into the following four categories: (1) COVID-19 prevalence (regarding the time), (2) COVID-19 attachment (cell connection of COVID-19), (3) COVID-19 in Hypertensive subjects (cellular and systematic mechanism of COVID-19 in subjects with hypertension), and (4) Exercise, COVID-19 and RAS system (the response of RAS to training and COVID-19 in cellular and systemic levels). Eventually, after the forenamed calcification and our prescriptions in this study, 68 articles were selected.

## Discussion

According to the past literature and the findings of previous research in the field of coronavirus and pharmacological and non-pharmacological strategies to prevent and treat this disease, in the following portion we will investigate multiple conceivable mechanisms. Based on Fang et al. (One of the most frequently corona-related studies), people with high blood pressure due to the use of ACE inhibitors and type 1 angiotensin receptor inhibitors (AT1) have higher expression of ACE2 (Coronavirus receptor at the cell surface) protein, and thus are more susceptible to SARS-COVID-2 (28,35,36). Moreover, regarding the increment expression of ACE2 protein due to compensatory mechanisms in hypertensive subjects to modify or neutralize the effects of the RAS (Renin-angiotensin system) axis, these subjects will have a premier risk of corona disease. 

At present time, new studies have provided evidences for the impact of ACE2 as a crucial factor in the entry of coronavirus into different organs such as the lungs and heart. It is supposed that athletes and people who get regular exercise due to axis amplification of the ACE2-Ang1-7-Mas receptor resulting in exercise-physiological adaptations, have higher levels of ACE2 and therefore probably higher chance of infection than healthy individuals (subjects without comorbidity)(37–40).

While the joint similarities between hypertensive patients and athletes or active subjects is an increase of ACE2 tissue expression and the serum levels resulting in disease related-compensatory mechanism and regular training (Pathophysiological and physiologically), respectively, there are plenty of differences in immune cells, proteins expression, and angiotensin2 levels and related-signaling pathways such as oxidative stress, inflammatory and apoptosis indices in organs that express ANG2 (22,37,41,42). 

But, in the treatment phase and COVID-19 consequences, the reports are contradictory; as a result, some studies have expressed the levels of serum ACE2 as a marker of this disease and its serum levels growth as a marker to recognize the severity of this disease (43–47). On the other side, other scholars indicated that angiotensin 2 is an indication of COVID-19 and its severity. At present, regarding the findings of Andre et al., as well as proving the affirmative inscription of the ACE2-Ang1-7-Mas receptor axis in controlling and suppressing inflammation and pulmonary fibrosis (27), we hypothesized that the increment of serum ACE2 concentration is induced by enhancement of pulmonary expression of this protein and also the body's compensatory mechanisms to prohibiting of the destructive effects of angiotensin 2 and related organs such as lungs and heart (48,49).

The above mechanism can be one of the reasons for the recovery of COVID-19 cases (with or without hypertension) with the treatment of antihypertensive medications like ACE inhibitors and type 1 angiotensin receptor inhibitors because these drugs enforce to gained ACE2 expression and the ACE2-Ang1-7-Mas receptor axis, which is related with diminished expression and effects of angiotensin 2 in tissues such as the lungs and heart (28,50). Therefore, according to the above-mentioned mechanisms, we concluded that athletes and people who exercise and do physical activity regularly, due to the high concentration of ACE2 and lower levels of angiotensin 2 resulting in the exercise adaptation in these individuals, indicate less complications and severity symptoms of SARS-COV 2 such as dyspnea, hospitalization and heart disease (51).

**Figure 1:**
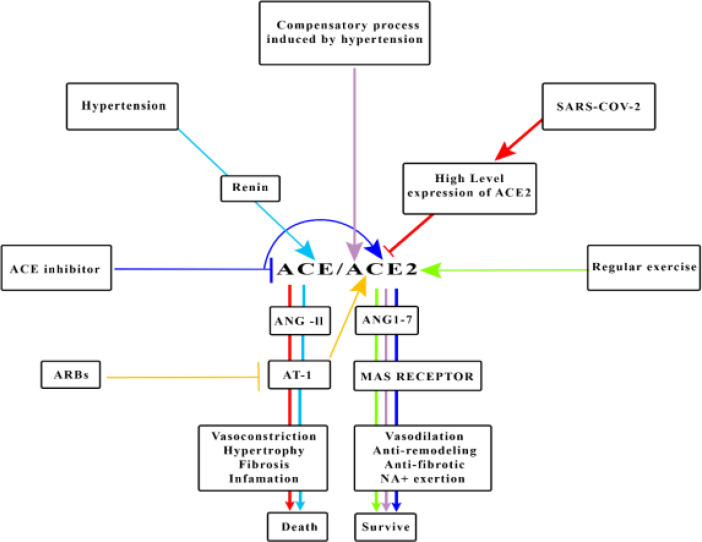
The pathways that are involved in COVID-19 disease in hypertensive patients that can lead to severe outcomes and death. On the contrary, the possible role of regular exercise in reducing the severity and COVID-19 mortality in active people are shown in Figure 1. ACE: Angiotensin-converting enzyme, ACE2: Angiotensin-converting enzyme 2, ARBs: Angiotensin II receptor blockers, AT1: Angiotensin II receptor type 1

In conclusion altogether, as it is shown in Figure 1, it can be concluded that athletes and hypertensive patients are more susceptible to COVID-19 because of high level of ACE2 as a result of physiologic and pathophysiologic process, but there are many differences such as severity, treatment phase as well as outcome-related SARS-COV2 between them.

**Suggestion: **On the whole, it can be declared that studies which focus on exercise and physical activity by sports physiologists can greatly indicate the role of physical activity and exercise in the prevention and treatment of SARS-COV2. For this reason, we suggest that sports physiology researchers be counseled in the case of required equipment, examining the differences in the severity of the disease, symptoms, and quarantine period between athletes and normal subjects because it can greatly determine the role of exercise in this disease as a preventive or strengthening factor. 

## Conflict Of Interest:

The authors declare no conflict of interest, financial or otherwise.
